# Gain-of-function miRNA signature by mutant p53 associates with poor cancer outcome

**DOI:** 10.18632/oncotarget.7090

**Published:** 2016-01-31

**Authors:** Yao Zhang, Ye Hu, Jing-Yuan Fang, Jie Xu

**Affiliations:** ^1^ State Key Laboratory for Oncogenes and Related Genes, Key Laboratory of Gastroenterology and Hepatology, Ministry of Health, Division of Gastroenterology and Hepatology, Renji Hospital, School of Medicine, Shanghai Jiao Tong University, Shanghai Institute of Digestive Disease, Shanghai Cancer Institute, Shanghai, China; ^2^ Xinhua Hospital, Shanghai Jiao Tong University School of Medicine, Shanghai 200092, China

**Keywords:** cancer prognosis, miRNA, mutation, non-negative matrix factorization, p53

## Abstract

Missense mutation of p53 not only impairs its tumor suppression function, but also causes oncogenic gain of function (GOF). The molecular underpinning of mutant p53 (mutp53) GOF is not fully understood, especially for the potential roles of non-coding genes. Here we identify the microRNA expression profile (microRNAome) of mutp53 on Arg282 by controlled microarray experiments, and clarify the prognostic significance of mutp53-regulated miRNAs in cancers. A predominant repression effect on miRNA expression was found for mutant p53, with 183 significantly downregulated and only 12 upregulated miRNAs. Mutp53 and wild-type (wtp53) commonly upregulate let-7i, and other two miRNAs were upregulated by wtp53 but repressed by mutp53 (miR-610 and miR-3065–3p). Based the mutp53-regulated miRNA signature, a non-negative matrix factorization (NMF) model classified gastric cancer (GC) cases into subgroups with significantly different Disease-free survival (Kaplan-Meier test, *P* = 0.013). In contrast, the NMF model based on all miRNAs did not associate with cancer outcome. The mutp53 miRNA signature associated with the outcomes of breast cancer (*P* = 0.024) and hepatocellular cancer (*P* = 0.012). The miRPath analysis revealed that mutp53-suppressed miRNAs associate with Hippo, TGF-β and stem cell signaling pathways. Taken together, our results highlight a miRNA-mediated GOF mechanism of mutant p53 on Arg282, and suggest the prognostic potential of mutp53-associated miRNA signature.

## INTRODUCTION

The TP53 gene that encodes the p53 tumor suppressor protein is the most commonly mutated gene in all human cancers [1], and missense mutation causing substitution of single amino acid represents the major type of mutations of TP53 gene [2]. The wild-type p53 is a master regulator of human genomic integrity [3], which is stabilized an accumulated in the nucleus in response to genomic stress or oncogenic signaling [4]. The p53 protein was firstly recognized as a transcription factor, which binds to DNA in a sequence-specific manner in its tetrameric form. The regulation of p53 monomer-tetramer assembly is regulated by c-abl and RhoGAPs [5]. The downstream target genes of p53 are involved in multiple pathways such as cell cycle arrest, apoptosis, and metabolism [6]. The cytoplasmic function of p53 protein associates with mitochondria outer membrane, where it binds BCL-XL and induces cytochrome C release and initiates apoptosis [7].

Missense mutation of p53 not only causes loss of tumor suppression function (LOF), but also causes gain of oncogenic function (GOF) [8]. Evidence based on both transgenic mouse model and human cancer data consistently support the GOF effect of mutant p53 (mutp53). When compared to p53-null mice [9], mice harboring the hot-spot mutant p53 displayed more spontaneous tumors and shorter survival. In addition, different p53 mutants have been confirmed to associate with different outcomes [10]. In a previous study, we have demonstrated that the hot-spot p53 mutation on Arg282 (R282) associated with significantly earlier cancer onset in Li-Fraumeni families that carry germline p53 mutations [11]. Moreover, the R282 mutation also associated with shorter survival of cancer patients, as reported in our previous study. Due to the variable signaling pathways and clinicopathological features of different p53 mutations, it has been proposed that p53 mutations should be considered as different oncogenes and biomarkers in cancers [12]. In a structural perspective, the R282 mutation may destabilize of the protein structure [13], induce protein aggregation [14], and affect its binding to BCL-XL in mitochondria [15]. However, the signaling pathways associated with R282 mutant are largely unknown, presenting a major challenge for developing targeted therapy.

MicroRNAs (miRNAs) are a class of non-coding RNAs with 20–25 nucleotides in length that function in RNA silencing and post-transcriptional regulation of gene expression [16] The miRNA expression profile (miRNAome) is significantly altered in cancers [17–19], and it is known that miRNAs may play causative roles in cancers by regulating multiple signaling pathways [20, 21]. The competing endogenous RNA (ceRNA) regulatory networks involving protein-coding messenger RNAs, long-noncoding RNAs and miRNAs are emerging factors that may contribute to cancer development [22, 23]. The miRNAome may be affected by multiple factors in cancer, including gene copy alteration [24, 25], transcription factor dysregulation, and the dynamics of ceRNA network. Besides, microRNAs have been generally accepted to be involved in the p53-regulated network, like contributing to the down-regulations of mRNA and protein expression observed after p53 activation [26]. Otherwise, we found previously that mutp53 R248W could transactivate the GAPLINC long non-coding RNA and promote the expression of CD44 oncogene [27]. Therefore, mutp53 GOF may not only involve protein-coding genes (PCGs) but also non-coding genes. Although some studies have already explored the correlations between cancers and miRNA, signature [28] and survival predictions [29], it is unknown to which extent mutp53 may affect the expression of miRNAs, and whether the mutp53 related miRNA signature may be prognostic in cancers.

In the present study, we characterize the mutp53 Arg282-regulated miRNAome in cancer cells, and analyze the prognostic significance of mutp53-regulated miRNA signature. The signaling pathways associated with the miRNA signature were analyzed with the miRPath algorithm, based on the enrichment of miRNA-mRNA target pairs in KEGG pathway. Through these approaches, we aim to identify the miRNA-mediated GOF mechanisms of Arg282 hotspot p53 mutation.

## RESULTS

### Identification of mutp53 R282W regulated miRNAs

**Figure 1:**
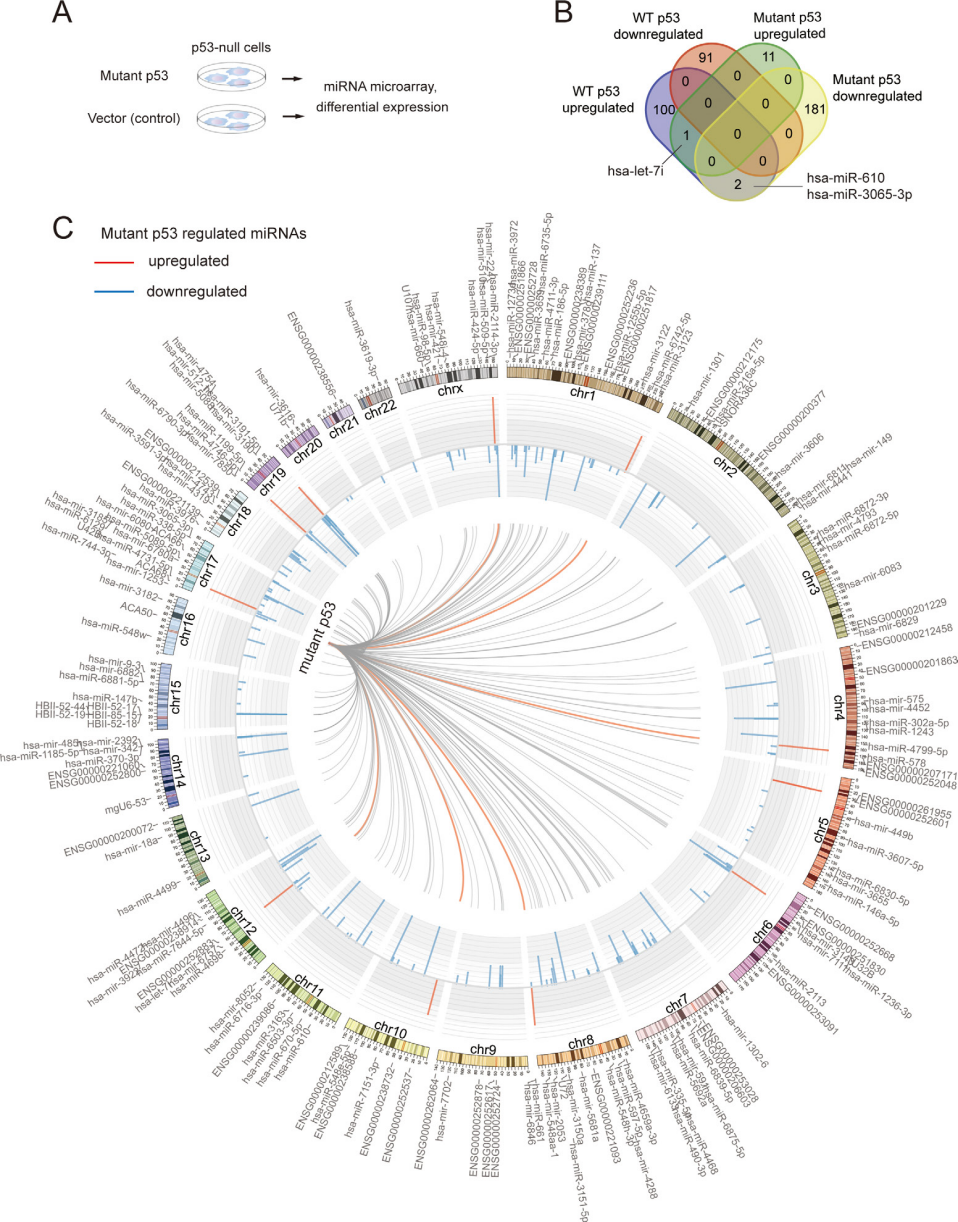
The miRNAome of mutp53 largely differs from that of wtp53 (**A**) Schematic representation of the study procedures. Mutant p53 or empty vector was stably transfected to p53-null H1290 cancer cells, followed by Affymetrix miRNA 4.0 microarray study and differential expression analysis. (**B**) Venn's diagram showing the common and unique miRNAs regulated by either mutp53 or wtp53. (**C)** The Circos map indicates the miRNAs that were upregulated (red) or downregulated (blue) by mutp53 R282W. The chromosomal locations of regulated miRNAs are also indicated.

To identify mutp53 R282W regulated miRNAs in an unbiased manner, we performed stable transfection of the p53-null H1299 cells with an expression vector encoding the p53 R282W mutant. After total RNA isolation, microarray assay (Affymetrix miRNA 4.0) was used to measure the expression profile of miRNAs. The cells transfected with empty vector was used as control, and each experimental group had triple biological repeats (schematics of experimental procedures shown in Figure 1A). Because fold-change ranking combined with a non-stringent statistical *P*-value [30–32] has been found to be more reliable for microarray-based differential expression analysis, we used the widely-accepted criteria of fold-change *>* 2 (or *<* 0.5) and *P*-value *<* 0.05 [33–36]. Interestingly, we found that mutant p53 R282W had a predominant repression effect on miRNA epxression, with 183 downregulated miRNAs (Supplementary Tables S1) but only 12 upregulated miRNAs (Supplementary Tables S2). We have already uploaded the microRNA data to the GEO website (Series GSE 73876).

### Most mutp53-regulated miRNAs are unrelated to wtp53

We further analyzed if mutp53 R282W-regulated miRNAs might partially overlap with those regulated by the wild-type p53 (wtp53). The wtp53-regulated miRNAs were determined based on the raw data of a recently published paper, using the same criteria as mutp53 [37]. Interestingly, only one miRNA, namely hsa-let-7i was found commonly upregulated by wtp53 and mutp53. Two other miRNAs (hsa-miR-610 and hsa-miR-3p) were downregulated by mutp53, but upregulated by wtp53. No miRNA was found commonly downregulated by mutp53 and wtp53 (Figure 1B). Of note, the mutp53 regulated miRNAs were found with uneven chromosomal distribution. While chromosome 1 contained 21 miRNAs that were regulated by mutp53, chromosomes 21 and 22 only contained one such miRNA, respectively (Figure 1C, listed in Supplementary Table S3). The vast majority of mutp53 regulatory targets were unrelated to wtp53, suggesting that mutp53 may acquire GOF effects through regulating miRNAs.

### Mutp53-regulated miRNA signature associates with prognosis of gastric cancer

**Figure 2:**
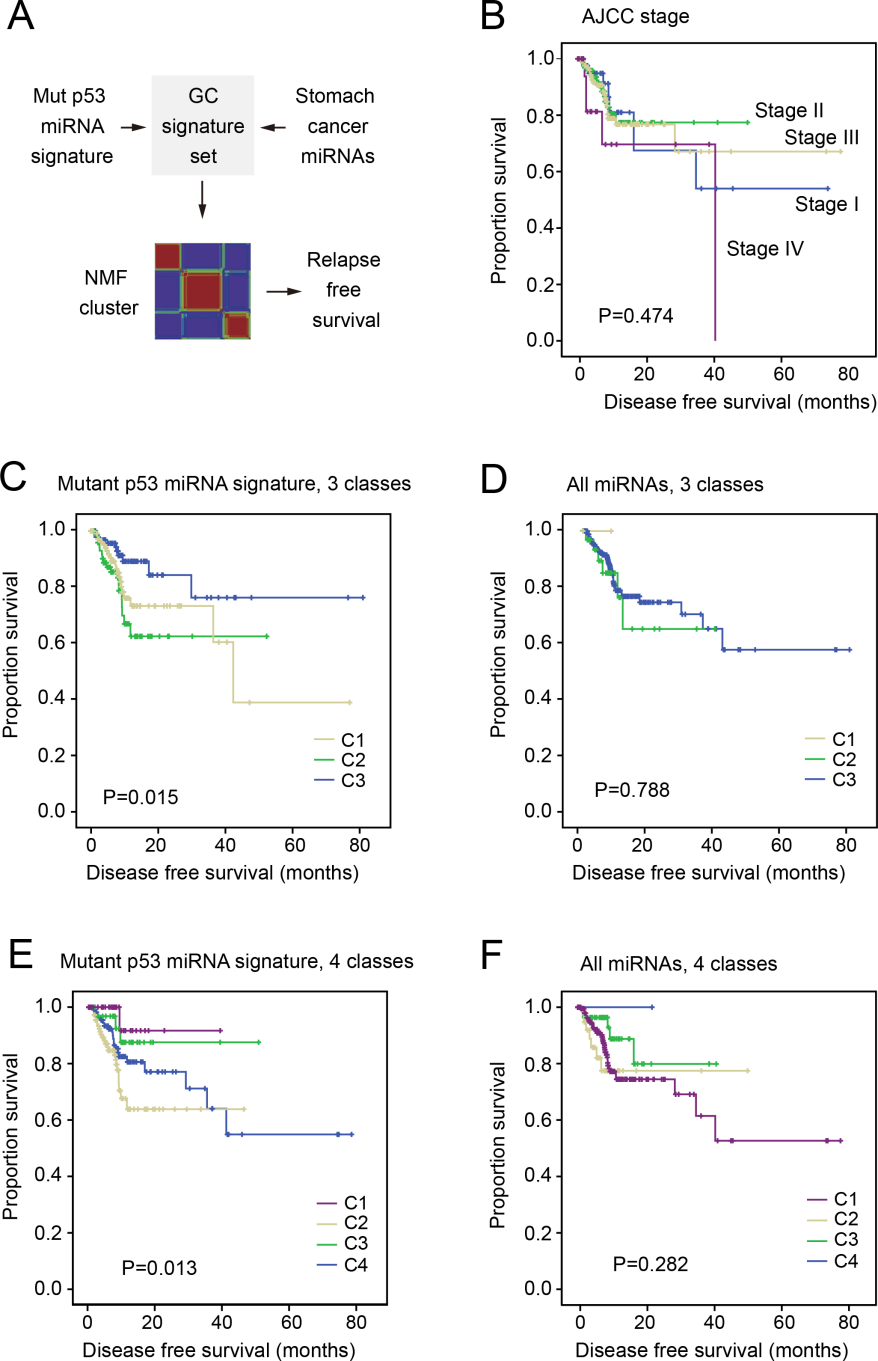
The miRNA signature of mutp53 associated with disease-free survival of gastric cancer patients Comparison of survival in all panels were performed using Kaplan-Meier test. (**A**) The schematic diagram showing the analysis flow. A partial mutp53 signature was derived by overlapping mutp53-regulated miRNAs and abundantly expressed miRNAs in gastric cancer. a non-negative matrix factorization (NMF) model was used to classify the cancer patients based on the miRNA signature, followed by DFS comparison between different groups. (**B**) The survival of gastric cancer patients with different AJCC stages were compared using Kaplan-Meier survival test (*P*-value indicated). (**C–D**) DFS of patients in 3 subgroups determined by NMF clustering based on either the miRNA signature (C) or all miRNAs (D). (**E–F**) Comparison of patient DFS in 4 subgroups as determined based on miRNA signature (E) or all miRNAs (F).

**Figure 3:**
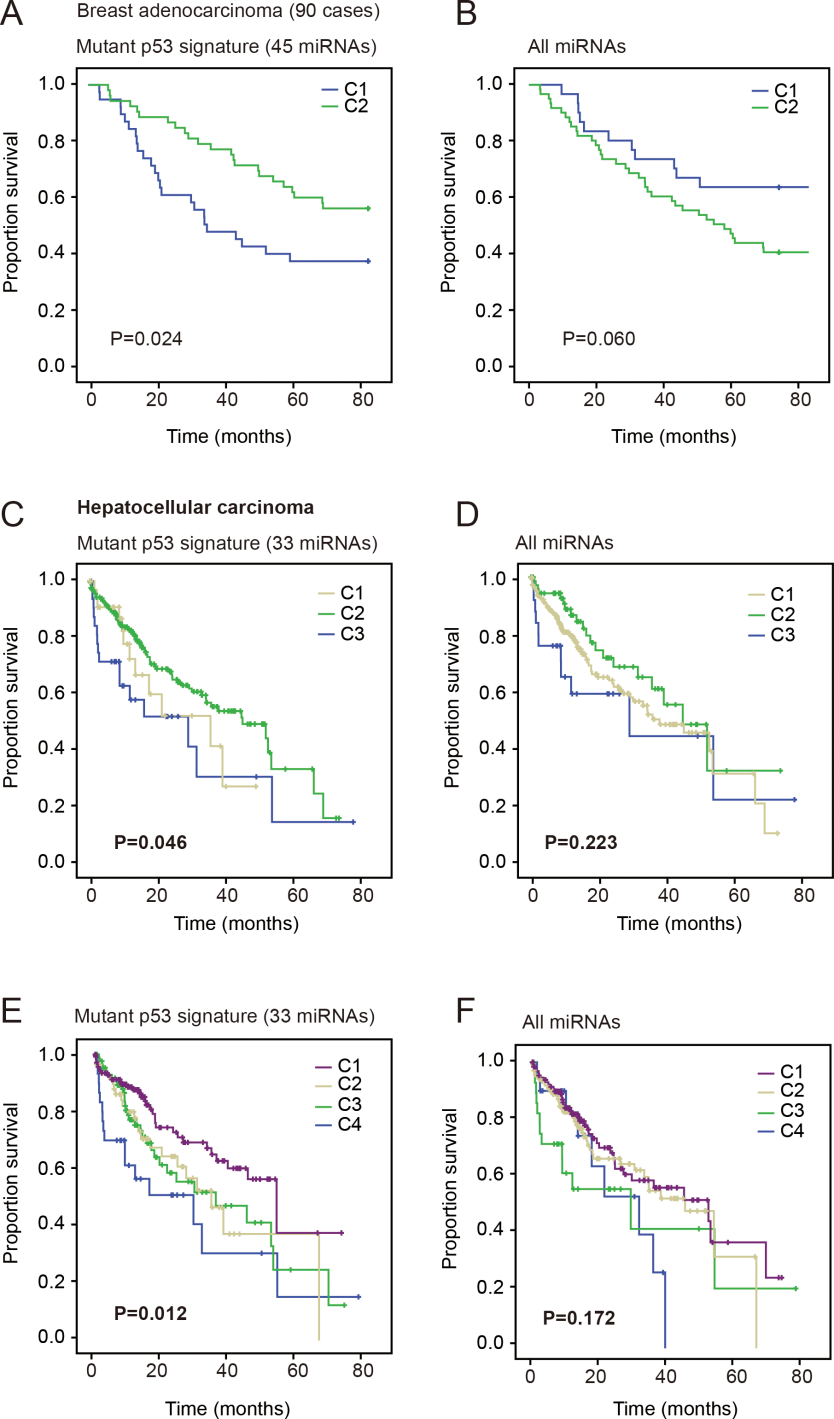
The mutp53 miRNA signature associates with prognosis of breast and liver cancers Comparison of survival in all panels were performed using Kaplan-Meier test. (**A–B**) Disease-free survival of breast cancers with lymphnode involvement. The subgroups determined by miRNA signature displayed significantly different DFS (A), but the subgroups determined by all miRNAs did not significantly associate with DFS (B). (**C–D**) Survival of patients in 3 subgroups of liver cancer patients classified by the mutp53 miRNA signature (C) or all miRNAs (D). (**E–F**) The comparison of patient survival in 4 subgroups determined based on miRNA signature (E) or all miRNAs (F).

We further questioned whether the mutp53-regulated miRNAs may associate with the prognosis of cancers. The expression profiles of miRNAs were obtained from the gastric adenocarcinoma dataset of the cancer genome atlas (TCGA) that included 470 cancer cases [38]. Among the 195 mutp53-regulated miRNAs, 38 were found abundantly expressed in the gastric cancer samples (list in Supplementary Table S4). This partial mutp53 signature containing 38 miRNAs was then used to calculate the similarity between cancer samples (schematic diagram in Figure 2A). Since the non-negative matrix factorization (NMF) model has been successfully applied to classify cancer samples with clinical significance [39], we also employed the NMF method in the present study (detailed procedures described in the Methods section). Interestingly, the partial mutp53 miRNA signature classified the cancer samples into 3 or 4 subgroups with significantly different disease-free survival (*P =* 0.015 and *P =* 0.013, respectively). In contrast, NMF clustering revealed subgroups with no association with cancer outcome (Figure 3).

Of note, the miRNA signature-based classification displayed stronger association with cancer outcome than the traditional AJCC staging (Figure 3) and microsatellite instability (Supplementary Figure S1). When the p53 gene status was categorized as wild type, missense mutation or truncating mutation, patients with different p53 status displayed no significant difference in DFS. Likewise, the p53 gene copy number, mRNA expression, and protein expression displayed no significant association with DFS (Supplementary Figure S1). These may be due to the complexity of p53 regulation at genetic, epigenetic, and protein levels. Thus, one such factor alone may not perfectly mark its functional status.

### Mutp53 miRNA signature in the prognosis of breast and liver cancers

Since p53 is also frequently mutated in multiple cancer types, we also examined the association of mutp53 miRNA signature with the prognosis of other cancers. The breast cancer dataset reported by Buffa and colleagues [40] contained both miRNA expression profile and patient DFS, and thus was included in this study. The NMF model divided the patients into 2 subgroups, because more groups caused significant decrease in cophenetic correlation. As a result, the partial mutp53 signature (listed in Supplementary Table S4) classified breast cancer patients with significantly different DFS (*P =* 0.024, Kaplan-Meier survival test). In contrast, the classification model based on all miRNAs showed no significant association with DFS (*P =* 0.06, Figure 3A and 3B).

Further, we analyzed the prognostic effect of mutp53 miRNA signature in liver cancer. Since the disease-free survival data of the TCGA liver cancer patients were more incomplete (247 of 377 cases unavailable), we analyzed the overall survival of patients. Again, the partial mutp53 signature (miRNAs listed in Supplementary Table S4) divided patients into 3 or 4 groups with significantly different survival (*P =* 0.046 and *P =* 0.012), but the model based on all miRNAs displayed no significant association (Figure 3C–3F). These results consistently suggest that mutp53 miRNA signature is associated with cancer outcome.

### Mutp53-repressed miRNAs target multiple cancer-related pathways

**Figure 4:**
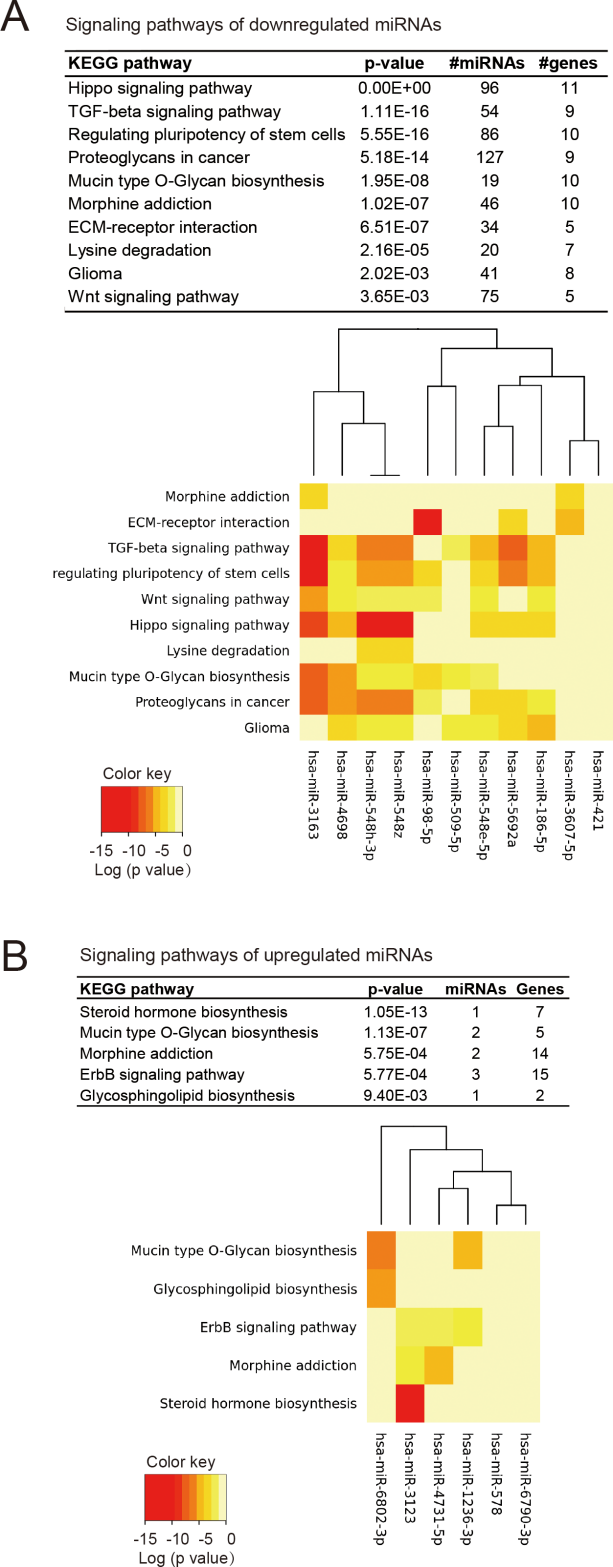
The signaling pathways associated with mutp53-regulated miRNAs (**A**) KEGG signaling pathways that significantly associated with miRNAs repressed by mutp53. The results were based on miRPath, an experimentally supported tool for identifying miRNA-targeted pathways. The significance, number of targeted genes and miRNAs are shown in the upper panel, and the heat map in the lower panel indicate the involvement of top-ranked miRNAs in the indicated signaling pathway. (**B**) The pathway enrichment analysis of mutp53-upregulated miRNAs. The significance of each pathway and the association of top-ranked miRNAs are respectively shown in the upper and lower panels.

To probe the signaling pathways of mutp53-regulated miRNAs, we performed pathway enrichment analysis for miRNAs using the miRPath algorithm, which is based on more than 600,000 experimentally supported miRNA targets from DIANA-TarBase [41]. Since the mutp53 displayed predominant suppression effect on miRNAs, we firstly analyzed the pathways that were targeted by the downregulated miRNAs (in positive association with mutp53 function). The hippo signaling pathway was the most significantly associated gene set, containing 11 genes that were targeted by 96 miRNAs (Figure 4A). These miRNAs represent half of all mutp53-repressed miRNAs, indicating a strong association between mutp53 and the Hippo signaling pathway. The pathways belonging to TGF-β signaling, regulation of pluripotency of stem cells, proteoglycans in cancer were also significantly associated with mutp53-repressed miRNAs (Figure 4A). The miRNAs upregulated by mutp53 were associated with specific metabolic pathways (steroid hormone, mycin type O-glycan, and glycosphingolipid biosynthesis), as well as the ErbB signaling (Figure 4B). Note that such associations were less significant, due to the smaller number of miRNAs upregulated by mutp53. In fact, the hippo signaling pathway has been reported to regulate the pluripotency of stem cells [42] and to interplay with the TGF-β pathway [43, 44], thus the pathways associated with mutp53-repressed miRNAs might reflect the significant involvement of hippo signaling.

## DISCUSSION

The mechanisms of mutp53 gain-of-function effect are important for developing targeted therapies against advanced cancers, and our study presents the first unbiased characterization of mutp53 R282W-regulated miRNAs. We demonstrate that a mutp53 miRNA signature can identify cancer subgroups with significantly different outcomes, and the hippo signaling is associated with the mutp53 signature.

Firstly, our data revealed a novel miRNAome of mutp53 that substantially differs from that of wtp53. Among the 195 mutp53-regulated miRNAs, only 3 were related to wtp53. As reported previously, the wtp53 regulates a broad panel of miRNAs with potential tumor suppressing roles, including miR-34a-5p, miR-182–5p, miR-203a, miR-222–3p, and miR-432–5p, etc [45–47]. Our data suggest that on a p53-null background, ectopic expression of mutp53 repressed the expression of many miRNAs that are not regulated by wtp53. This may be explained by the two major GOF models: 1) mutp53 may bind new gene promoters and acquire a novel transcriptome; 2) mutp53 may interact with other proteins and indirectly regulate gene expression through its mutant interactome [12, 48]. Since miRNAs belong to competing endogenous RNA (ceRNA) networks, the intermediate pathways between mutp3 and miRNA expression might be complicated. It deserves further investigation whether other coding genes or long noncoding genes might be involved in the effects of mutp53 on miRNA expression.

Moreover, our results demonstrate that a mutp53 GOF signature composed of miRNAs could successfully divide patients into subgroups with clinical relevance. It has been suggested that GOF mutation of p53 associates with worse cancer outcomes, but our results revealed for the first time that mutp53-regulated miRNAs have prognostic significance. Indeed, the miRNA signature displayed stronger association with gastric cancer DFS, over p53 genetic status or its expression level. The expression of miRNAs were strongly influenced by mutp53, and thus a miRNA signature could reflect the functional status of p53. Other factors such as p53 genetic status, copy number alteration, mRNA level, or protein level were not ideal markers for p53 function, which is actually a result of interaction between the above-mentioned factors. It should be noted that miRNA expression profiles differ substantially between cancer types, thus the miRNA signatures for gastric, liver and breast cancers were distinct subsets of the mutp53-regulated miRNAs. Even though, the partial mutp53 signature displayed prognostic significance in these cancer types.

Finally, our pathway analysis revealed that mutp53 R282W associated with the hippo signaling, which is involved in regulating pluripotency of stem cells and cancer aggressiveness [49, 50]. The experimentally supported miRPath algorithm identified multiple genes in the hippo pathway that could be targeted by miRNAs in the mutp53 signature, suggesting that mutp53 R282W may acquire GOF effect through this oncogenic route. This finding also raises the possibility of targeting hippo signaling pathway in cancer cases bearing the p53 R282 hot-spot mutation.

In conclusion, the hot-spot mutp53 R282W regulates a novel miRNAome that largely differs from that of wtp53, and this mutant GOF signature associates with poor prognosis of multiple cancer types. In future research, the mechanisms underlying this mutant-specific miRNAome should be investigated, which may facilitate the development of more effective targeted cancer therapies.

## METHODS

### Plasmid construction

The pcDNA3-HA-p53 expression vector was constructed by inserting PCR-amplified p53 cDNA sequence into pcDNA3 vector (Invitrogen, Carlsbad, CA, USA). The p53 R282W mutant was derived by site-directed mutagenesis PCR reaction using platinum PWO SuperYield DNA polymerase (Roche, Basel, Switzerland) according to the product manual. The plasmid was sequenced to confirm if the designed mutation is present, without any other unwanted mutation.

### Cell culture and transfection

The human H1299 (p53-null) cells were maintained in DMEM medium (Gibco, Gaithersburg, MD, USA) supplemented with 10% fetal bovine serum (Invitrogen) and cultured in a humidified incubator at 37°C under 5% CO2. Before transfection, cells were seeded into normal growth medium at 50% confluence in six-well tissue plates. The FuGENE HD transfection reagent (Promega, Fitchburg, WI, USA) was applied according to the product manual. Briefely, the transfection complex was made by 1 μg plasmid, 3 μl FuGENE HD and 100 μl media. Six hours after the complex was added to the cells, normal culture media was used to culture cells for additional 48 h, followed by gene expression analysis. For stable transfection, the 600 μg/mL G418 was used to culture cells for four weeks, and the expression of mutp53 in stable strains was confirmed by quantitative PCR.

### MicroRNA-microarray experiment

The H1299 cells stably transfected with mutp53 R282W or the control vector were respectively analyzed by Affymetrix GeneChip miRNA 4.0 microarray with three biological replications. A total of 200 ng small RNA was used in sample preparation with a FlashTag Biotin RNA Labeling Kit for Affymetrix GeneChip miRNA arrays (Genisphere). The labeled RNA was consequently hybridized for sixteen hours to an Affymetrix GeneChip miRNA array according to the product manual. Microarrays were washed and stained in the Affymetrix Fluidics Station 450, and scanned on the Affymetrix G3000 GeneArray Scanner. The image files were analyzed using the Affymetrix software (Expression Console), Robust Multi-array Average (RMA) background correction, log-2 transformations and global normalization methods were performed for data pre-processing, and normalization.

### Unsupervised clustering by non-negative matrix factorization (NMF) algorithm

NMF is a matrix factorization algorithm that focuses on the analysis of data matrices whose elements are nonnegative, and the principles and the detailed algorithm has been described previously [51]. We employed the NMF algorithm module of the MEV 4.9 program package [52] to perform unsupervised clustering of cancer samples, with «divergence» as the update rule and maximum iteration of 1,000. The optimal number of classes was determined according to the cophenetic correlation value, as described in our previous study [39].

### Survival analysis

The survival analysis was carried out using the SPSS software package. In the Kaplan–Meier (log rank) survival test model, the censored status was indicated when the patient was still alive (or cancer-free) at the time of follow-up. The criteria of *P* < 0.05 was used for judging statistical significance.

### Pathway analysis of miRNAs

The signaling pathways in association with miRNAs were analyzed with the miRPath v3.0 web server as described previously [41]. Briefly, the down-regulated (or up-regulated) miRNAs in the mutp53 signature were used as input, and the microT-CDS method was used for determining target genes. The species was defined as “human”, and the gene filter was set as “determine genes”. The enrichment of target genes in different KEGG molecular pathways were ranked by the respective *P*-values (adjusted by false discovery rate, FDR). The criteria of *P* < 0.01 (adjusted) was used for judging statistically significance.

## SUPPLEMENTARY MATERIALS FIGURE AND TABLES




