# Supplementary material for: Gain-of-function miRNA signature by mutant p53 associates with poor cancer outcome
Source: Oncotarget. 2016 Jan 31;7(10):11056–66. doi: 10.18632/oncotarget.7090 (PMC4905457; doi:10.18632/oncotarget.7090)
Supplement: Supplementary file 1 [file oncotarget-07-11056-s001.pdf]

Gain-of-function miRNA signature by mutant p53 associates with poor cancer outcome

Supplementary Materials

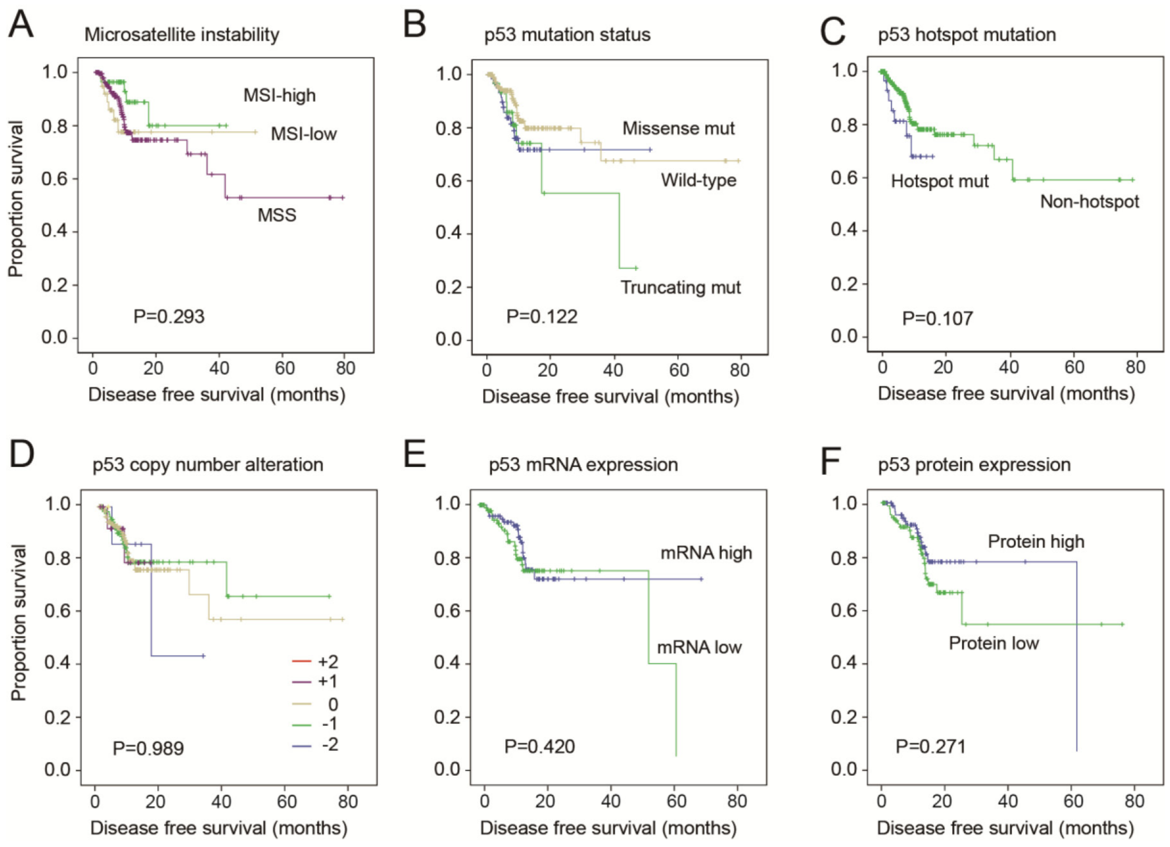

Supplementary Figure S1: Association of MSI, p53 genetic status and expression with DFS of gastric cancer. The survival of patients in different groups were compared with the Kaplan-Meier survival test. The criteria of $P < 0.05$ was used to judge statistical significance.

Supplementary Table S1: The miRNAs that were downregulated by mutp53 R282W (FC < 0.5, $P < 0.05$)

Supplementary Table S2: Upregulated miRNAs by mutp53 R282W (FC < 0.5, *P* < 0.05)

miRNA_gene	control.rep1	control.rep2	control.rep3	mutant_p53.rep1	mutant_p53.rep2	mutant_p53.rep3
hsa-mir-578	–0.1023048	0.00385242	–0.1035968	0.0927383	0.1921451	0.2258548
ENSG00000252601	0.02464659	–0.08982608	–0.07201257	0.04046368	0.1188284	0.1356783
ENSG00000261955	0.02464659	–0.08982608	–0.07201257	0.04046368	0.1188284	0.1356783
hsa-miR-4731-5p	–0.1770857	0.05409777	–0.1966194	0.3404264	0.08984698	0.2125069
hsa-miR-6790-3p	–0.2140777	0.01669325	–0.2377214	0.3404264	0.407616	0.06386299
hsa-mir-510	0.009101495	0.07270514	0.09308218	0.2437083	0.2635161	0.1592286
hsa-miR-1236-3p	–0.01649719	–0.1973925	–0.09133096	0.1798379	0.2125069	0.04645495
hsa-mir-3123	–0.02753729	0.1626393	0.31572	0.6190575	0.4776919	0.388635
hsa-miR-6802-3p	–0.07787399	–0.169787	–0.08024121	–0.008114949	0.2187319	0.1922264
hsa-mir-6846	–0.1535122	–0.1535122	–0.1535122	0.1353019	0.2242937	0.1889334
hsa-let-7i	–0.11206	–0.05207927	–0.2969556	0.2303855	0.07145564	0.2215828
ENSG00000252537	–0.09302633	–0.03305989	–0.3335435	0.08233424	0.1755946	0.2494192

Normalized expression levels in three experimental repeats are shown in the table.

Supplementary Table S3: Common and unique miRNAs that were regulated by mutp53 and/or wtp53

Features	Number	List of miRNAs
Mut_p53_upregulated & WT_p53_upregulated	1	hsa-let-7i
Mut_p53_downregulated & WT_p53_upregulated	2	hsa-miR-610<br>hsa-miR-3065–3p
WT_p53_upregulated	100	hsa-miR-193a-3p hsa-miR-3937 hsa-miR-877 hsa-miR-1266 hsa-miR-138–1 hsa-miR-548s hsa-miR-1468 hsa-miR-22 hsa-miR-127–3p hsa-miR-625 hsa-miR-592 hsa-miR-192 hsa-miR-766 hsa-miR-570 hsa-miR-940 hsa-miR-1294 hsa-miR-222 hsa-miR-210 hsa-miR-3168 hsa-miR-125a-3p hsa-miR-193a-5p hsa-miR-195 hsa-miR-548j hsa-miR-549 hsa-miR-3605–3p hsa-miR-1304 hsa-miR-629 hsa-miR-3613–3p hsa-miR-3174 hsa-miR-491–3p hsa-miR-335 hsa-miR-452 hsa-miR-1274a hsa-miR-362–3p hsa-miR-33b hsa-miR-1229 hsa-miR-3663–5p hsa-miR-143 hsa-miR-142–5p hsa-miR-3197 hsa-miR-3162 hsa-miR-301a hsa-miR-449a hsa-miR-141 hsa-miR-203 hsa-miR-361–3p hsa-miR-194 hsa-miR-18a hsa-miR-205 hsa-miR-224 hsa-miR-185 hsa-miR-3938 hsa-miR-199a-3p hsa-miR-99b hsa-miR-483–5p hsa-let-7e hsa-miR-199a-5p hsa-miR-937 hsa-miR-1287 hsa-miR-339–5p hsa-miR-486–5p hsa-miR-576–5p hsa-miR-3617 hsa-miR-3925 hsa-miR-23a hsa-miR-891a hsa-miR-4286 hsa-miR-199b-3p hsa-miR-641 hsa-miR-577 hsa-miR-188–5p hsa-miR-652 hsa-miR-371–5p hsa-miR-324–3p hsa-miR-373 hsa-miR-200c hsa-miR-145 hsa-miR-130a hsa-miR-148b hsa-miR-3691 hsa-miR-362–5p hsa-miR-1914 hsa-miR-371–3p hsa-miR-573 hsa-miR-3164 hsa-miR-218 hsa-miR-372 hsa-miR-499–3p hsa-miR-188–3p hsa-miR-2355–3p hsa-miR-548u hsa-miR-29c hsa-miR-132 hsa-miR-3661 hsa-miR-215 hsa-miR-1293 hsa-miR-34c-3p hsa-miR-95 hsa-miR-34a hsa-miR-1976
WT_p53_downregulated	91	hsa-miR-140–5p hsa-miR-146a hsa-miR-502–3p hsa-miR-1538 hsa-miR-200b hsa-miR-32 hsa-miR-1254 hsa-miR-3191 hsa-miR-18b hsa-miR-3200–5p hsa-miR-1226 hsa-miR-1262 hsa-miR-125b hsa-miR-542–3p hsa-miR-3613–5p hsa-miR-219–1–3p hsa-miR-429 hsa-miR-508–3p hsa-let-7g hsa-miR-616 hsa-miR-374b hsa-miR-181a hsa-miR-184 hsa-miR-378c hsa-miR-2110 hsa-miR-221 hsa-miR-935 hsa-miR-636 hsa-miR-3928 hsa-miR-3130–5p hsa-miR-200a hsa-miR-30d hsa-miR-93 hsa-miR-3181 hsa-miR-3939 hsa-miR-23b hsa-miR-551a hsa-miR-330–3p hsa-miR-378 hsa-miR-191 hsa-miR-381 hsa-miR-3192 hsa-miR-150 hsa-miR-2277–5p hsa-miR-1908 hsa-miR-501–5p hsa-miR-574–3p hsa-miR-92a-1 hsa-miR-4326 hsa-miR-3909 hsa-miR-92b hsa-miR-130b hsa-miR-3120 hsa-miR-378b hsa-miR-339–3p hsa-miR-3130–3p hsa-miR-182 hsa-miR-615–3p hsa-miR-454 hsa-miR-30b hsa-miR-3188 hsa-miR-29b-1 hsa-miR-25 hsa-miR-2116 hsa-miR-365 hsa-miR-1305 hsa-miR-3200–3p hsa-miR-30a hsa-miR-20a hsa-miR-425 hsa-miR-3124 hsa-miR-627 hsa-miR-1 hsa-miR-1227 hsa-miR-196b hsa-miR-767–5p hsa-miR-340 hsa-miR-499–5p hsa-miR-3194 hsa-miR-484 hsa-miR-720 hsa-miR-664 hsa-miR-3131 hsa-miR-320d hsa-miR-3187 hsa-miR-590–3p hsa-miR-1275 hsa-miR-142–3p hsa-miR-3159 hsa-miR-589 hsa-miR-939
Mut_p53_upregulated	11	hsa-miR-6790–3p ENSG00000252601 hsa-miR-4731–5p hsa-mir-510 hsa-mir-3123 ENSG00000261955 ENSG00000252537 hsa-miR-6802–3p hsa-miR-1236–3p hsa-mir-578 hsa-mir-6846

Mut_p53_downregulated	182	hsa-miR-1185–5p ENSG00000212539 hsa-miR-1199–5p hsa-mir-149 ENSG00000221093 hsa-miR-1273d hsa-miR-1243 U32B hsa-mir-4793 ENSG00000252878 hsa-miR-3619–3p ENSG00000201863 hsa-miR-6503–3p hsa-mir-342 U107 ENSG00000206603 hsa-miR-4698 hsa-mir-3616 ENSG00000253091 hsa-miR-6807–3p hsa-miR-548w hsa-mir-224 ENSG00000238588 hsa-mir-449b hsa-mir-18a U42B ENSG00000221060 hsa-miR-5089–5p ENSG00000221139 ENSG00000212458 hsa-miR-6804–5p hsa-mir-660 ENSG00000238556 hsa-mir-2053 ACA66 hsa-miR-4256 hsa-miR-4468 hsa-miR-2114–3p hsa-miR-5692a hsa-mir-421 hsa-miR-3163 ENSG00000239086 hsa-miR-7151–3p hsa-miR-516b-3p hsa-mir-4743 hsa-mir-3606 HBI-100 hsa-miR-6839–5p hsa-mir-512–1 hsa-miR-3972 ENSG00000212589 hsa-miR-4659a-3p hsa-miR-146a-5p hsa-mir-3150a hsa-miR-6834–3p hsa-mir-6080 hsa-miR-6742–5p mgU6–53 hsa-mir-512–2 hsa-mir-6757 hsa-mir-6811 hsa-miR-490–3p hsa-miR-6881–5p hsa-miR-424–5p hsa-miR-3976 ENSG00000238732 hsa-miR-548e-5p hsa-miR-516a-3p hsa-miR-670–5p hsa-miR-661 ENSG00000251866 hsa-miR-6793–5p ENSG00000252724 hsa-miR-4799–5p ENSG00000207171 hsa-mir-5681a hsa-mir-548i-4 hsa-mir-2392 hsa-miR-4525 hsa-miR-7844–5p hsa-mir-378g hsa-miR-597–5p ENSG00000238935 hsa-miR-4472 hsa-miR-3191–5p hsa-miR-6872–3p hsa-miR-302a-5p ENSG00000201229 hsa-mir-4754 hsa-miR-4499 hsa-mir-3190 hsa-miR-370–3p hsa-mir-4319 hsa-miR-6716–3p hsa-mir-3182 ENSG00000252800 hsa-mir-591 hsa-mir-5088 hsa-miR-335–5p hsa-miR-6735–5p hsa-mir-6780a hsa-mir-3143 ENSG00000200377 ENSG00000238885 hsa-mir-7111 ENSG00000252133 hsa-miR-3607–5p hsa-mir-7702 ENSG00000200072 ENSG00000252617 hsa-miR-186–5p hsa-mir-1302–6 hsa-miR-6129 ENSG00000212175 ENSG00000252048 ENSG00000251830 hsa-mir-575 hsa-miR-338–5p hsa-mir-4496 ENSG00000253028 hsa-mir-548aa-1 hsa-mir-485 ENSG00000252728 hsa-miR-6872–5p hsa-mir-7850 ENSG00000251817 hsa-miR-548z HBII-52–19 hsa-mir-1253 HBII-52–17 ENSG00000238389 hsa-miR-137 hsa-mir-3922 hsa-mir-4452 hsa-mir-6083 ENSG00000262064 hsa-mir-6882 hsa-mir-6829 ENSG00000251878 hsa-mir-4288 hsa-miR-4746–5p ENSG00000252265 hsa-miR-4711–3p hsa-miR-3591–3p hsa-miR-3151–5p ACA50 ENSG00000239111 hsa-miR-2113 hsa-mir-1290 hsa-miR-548 h-3p ACA68 hsa-mir-8052 hsa-miR-509–5p HBII-52–18 hsa-miR-98–5p hsa-mir-4441 hsa-mir-6133 hsa-miR-3659 hsa-mir-1301 ENSG00000238690 hsa-miR-147b hsa-mir-3122 ENSG00000252883 hsa-mir-3655 hsa-miR-1255b-5p hsa-miR-3622b-5p ENSG00000238914 hsa-miR-216a-5p U71b ENSG00000252236 hsa-mir-9–3 HBII-85–15 U72 ENSG00000252668 SNORA36C hsa-miR-6830–5p hsa-mir-3185 hsa-miR-4653–3p hsa-miR-6875–5p HBII-52–44 hsa-miR-744–3p

Supplementary Table S4: The miRNAs in the partial mutp53 signatures of gastric, liver and breast cancers

	Partial miRNA signature of mutant p53 in stomach adenocarcinoma (*n* = 38)	Partial miRNA signature of mutant p53 in hepatocellular carcinoma (*n* = 33)	Partial miRNA signature of mutant p53 in breast carcinoma (*n* = 45)
Cancer-specific miRNAs	hsa-mir-149	hsa-mir-149	hsa-miR-149
	hsa-mir-18a	hsa-mir-18a	hsa-miR-18a
	hsa-mir-224	hsa-mir-224	hsa-miR-224
	hsa-mir-421	hsa-mir-421	hsa-miR-421
	hsa-mir-449b	hsa-mir-449b	hsa-miR-449b
	hsa-mir-510	hsa-mir-510	hsa-miR-510
	hsa-mir-578	hsa-mir-578	hsa-miR-578
	hsa-mir-660	hsa-mir-660	hsa-miR-660
	hsa-let-7i	hsa-let-7i	hsa-let-7i
	hsa-mir-1301	hsa-mir-1301	hsa-miR-126
	hsa-mir-3163	hsa-mir-3163	hsa-miR-137
	hsa-mir-3190	hsa-mir-3190	hsa-miR-154
	hsa-mir-342	hsa-mir-342	hsa-miR-181a
	hsa-mir-3606	hsa-mir-3606	hsa-miR-182
	hsa-mir-3616	hsa-mir-3616	hsa-miR-191
	hsa-mir-3922	hsa-mir-3922	hsa-miR-200a
	hsa-mir-485	hsa-mir-485	hsa-miR-202
	hsa-mir-512–1	hsa-mir-512–1	hsa-miR-302a
	hsa-mir-512–2	hsa-mir-512–2	hsa-miR-302b
	hsa-mir-548w	hsa-mir-548w	hsa-miR-302c
	hsa-mir-9–3	hsa-mir-9–3	hsa-miR-30a
	hsa-mir-1197	hsa-mir-1243	hsa-miR-30e
	hsa-mir-1246	hsa-mir-1290	hsa-miR-363
	hsa-mir-1264	hsa-mir-137	hsa-miR-373
	hsa-mir-1273d	hsa-mir-147b	hsa-miR-376a
	hsa-mir-1292	hsa-mir-2113	hsa-miR-378
	hsa-mir-1302–6	hsa-mir-3143	hsa-miR-380
	hsa-mir-145	hsa-mir-3182	hsa-miR-425
	hsa-mir-3122	hsa-mir-3655	hsa-miR-432
	hsa-mir-3123	hsa-mir-3659	hsa-miR-452
	hsa-mir-3128	hsa-mir-591	hsa-miR-454
	hsa-mir-346	hsa-mir-610	hsa-miR-490–3p
	hsa-mir-3649	hsa-mir-661	hsa-miR-493
	hsa-mir-448		hsa-miR-516a-3p
	hsa-mir-495		hsa-miR-517a
	hsa-mir-513c		hsa-miR-518c

	hsa-mir-548aa-1		hsa-miR-518e
	hsa-mir-885		hsa-miR-518f
			hsa-miR-519e
			hsa-miR-526b
			hsa-miR-575
			hsa-miR-591
			hsa-miR-610
			hsa-miR-661
			hsa-miR-9

The common miRNAs that appeared in all cancer types are presented separately.
